# Glycocalyx heparan sulfate cleavage promotes endothelial cell angiopoietin-2 expression by impairing shear stress–related AMPK/FoxO1 signaling

**DOI:** 10.1172/jci.insight.155010

**Published:** 2022-08-08

**Authors:** Robert P. Richter, Amit R. Ashtekar, Lei Zheng, Danielle Pretorius, Tripathi Kaushlendra, Ralph D. Sanderson, Amit Gaggar, Jillian R. Richter

**Affiliations:** 1 Division of Pediatric Critical Care Medicine, Department of Pediatrics;; 2 Program in Protease and Matrix Biology;; 3 Center for Injury Science;; 4 Division of Acute Care Surgery, Department of Surgery;; 5 Division of Molecular & Cellular Pathology, Department of Pathology;; 6 O’Neal Comprehensive Cancer Center;; 7 Division of Pulmonary, Allergy & Critical Care Medicine, Department of Medicine;; 8 Lung Health Center; and; 9 Department of Biomedical Engineering, University of Alabama at Birmingham, Birmingham, Alabama, USA.

**Keywords:** Vascular Biology, Endothelial cells, Glycobiology, Proteases

## Abstract

Angiopoietin-2 (Ang-2) is a key mediator of vascular disease during sepsis, and elevated plasma levels of Ang-2 are associated with organ injury scores and poor clinical outcomes. We have previously observed that biomarkers of endothelial glycocalyx (EG) damage correlate with plasma Ang-2 levels, suggesting a potential mechanistic linkage between EG injury and Ang-2 expression during states of systemic inflammation. However, the cell signaling mechanisms regulating Ang-2 expression following EG damage are unknown. In the current study, we determined the temporal associations between plasma heparan sulfate (HS) levels as a marker of EG erosion and plasma Ang-2 levels in children with sepsis and in mouse models of sepsis. Second, we evaluated the role of shear stress–mediated 5′-adenosine monophosphate–activated protein kinase (AMPK) signaling in Ang-2 expression following enzymatic HS cleavage from the surface of human primary lung microvascular endothelial cells (HLMVECs). We found that plasma HS levels peaked before plasma Ang-2 levels in children and mice with sepsis. Further, we discovered that impaired AMPK signaling contributed to increased Ang-2 expression following HS cleavage from flow-conditioned HLMVECs, establishing a paradigm by which Ang-2 may be upregulated during sepsis.

## Graphical abstract

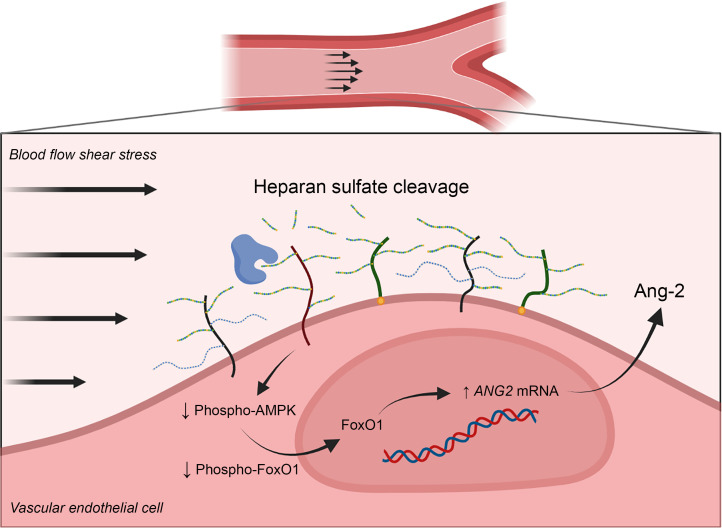

## Introduction

During the dysregulated host immune response that typifies sepsis (1), the vascular endothelium is systemically injured, culminating in organ dysfunction remote to the site of primary infection. Angiopoietin-2 (Ang-2) is an endothelium-derived cytokine expressed during inflammation that is now recognized as a key mediator of vascular dysfunction during sepsis (2, 3), and plasma levels of Ang-2 are associated with organ injury scores and clinical outcomes in children and adults with sepsis (4–6). While Ang-2 is a logical therapeutic target to abrogate sepsis-induced endothelial pathophysiology, the mechanisms driving Ang-2 expression during sepsis that would inform the development of effective treatment strategies have not been fully clarified.

The protein-rich glycocalyx that lines the luminal surface of blood vessels is essential to maintain homeostatic endothelial cell signaling and vascular function (7–9). Heparan sulfate proteoglycans (HSPGs) within the endothelial glycocalyx (EG) serve, among a host of functions, as transducers of apical surface shear stress to the endothelial intracellular environment affecting myriad downstream signaling pathways (10–14). Prior work suggests that the heparan sulfate (HS) side chains on EG HSPGs must remain intact to properly transmit surface shear stress to the endothelial intracellular environment (15, 16). Thus, enzymatic digestion of HS from the EG may contribute to deranged endothelial cell signaling during sepsis, including Ang-2 upregulation.

Heparanase is the only mammalian enzyme with activity on HS and is involved in sepsis-mediated lung, kidney, and intestinal injury through EG erosion (17–19). We previously observed that plasma levels of syndecan-1 HSPGs, indicating glycocalyx disruption, correlate with plasma Ang-2 levels following pediatric trauma (20), which may suggest a mechanistic linkage between EG injury and Ang-2 expression during systemic inflammatory states such as sepsis. Prior work demonstrates that Ang-2 protein expression in human umbilical vein endothelial cells (HUVECs) is suppressed by fluid shear stress via activation of 5′-adenosine monophosphate–activated protein kinase (AMPK) signaling (21). AMPK activation promotes the expression of the transcription factor Krüppel-like factor 2 (KLF2) and the inactivation of the transcription factor forkhead box O1 (FoxO1) (21, 22), both of which downregulate *ANG2* gene expression (23, 24).

In light of these clinical and preclinical observations, the objectives of the current study were first to establish the association between plasma HS levels and plasma Ang-2 levels in children with sepsis and in mouse models of sepsis and, second, to evaluate the role of HS as a mechanotransducer and regulator of Ang-2 expression via AMPK signaling. Herein, we report that plasma HS levels peaked prior to plasma Ang-2 levels in children and mice with sepsis. Our data also support the role of HS as a mechanoregulator of Ang-2 because we observed that enzymatic HS damage promoted an increase in Ang-2 expression from flow-conditioned human lung microvascular endothelial cells (HLMVECs), in part, through attenuation of AMPK signaling. Together, these findings suggest a potentially novel paradigm by which Ang-2 may be upregulated during sepsis via EG erosion and loss of HS-mediated mechanosignaling.

## Results

### Plasma HS levels peak prior to Ang-2 levels in human and murine sepsis.

**Figure 1:**
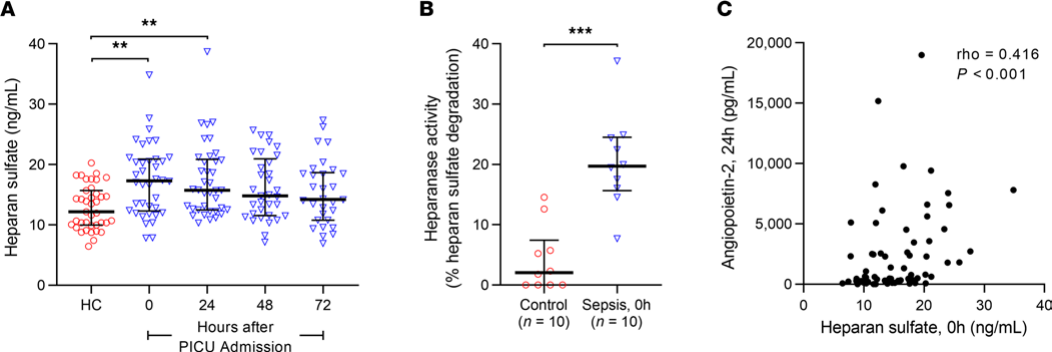
Plasma HS levels in children with sepsis peak at the time of PICU admission and correlate with plasma Ang-2 levels 24 hours after admission. (**A**) Plasma HS levels in children with sepsis at 0, 24, 48, and 72 hours following admission to the PICU compared with healthy controls (HC) (controls, *n* = 39; sepsis, 0 hours and 24 hours *n* = 38, 48 hours *n* = 33, 72 hours *n* = 30). **P < 0.01, versus control. (**B**) Plasma levels of heparanase activity were significantly elevated at PICU admission in 10 randomly chosen patients with sepsis compared with 10 randomly selected controls, corresponding with the elevations in plasma HS levels in children with sepsis at the time of PICU admission. ***P < 0.001. (**C**) Spearman’s rank-order correlation between plasma HS levels measured at PICU admission (0 hours) and plasma Ang-2 levels 24 hours after PICU admission in children with sepsis. Data are presented as medians with interquartile ranges and analyzed by ordinary 1-way Kruskal-Wallis ANOVA corrected by Dunn’s multiple comparisons test (**A**) or by Mann-Whitney *U* test (**B**).

We have previously demonstrated that plasma Ang-2 levels are elevated in children with sepsis and peak by 24 hours after pediatric intensive care unit (PICU) admission before a gradual decline (4). In the same cohort, we observed that plasma HS levels were also elevated at the time of admission to the PICU compared with healthy pediatric controls, peaking at PICU admission (Figure 1A). This finding was consistent with increased plasma heparanase activity levels at PICU admission (Figure 1B). Further, HS levels at admission correlated with plasma levels of Ang-2 24 hours following PICU admission (Figure 1C), demonstrating a temporal association between EG damage prior to elevated Ang-2 expression.

**Figure 2:**
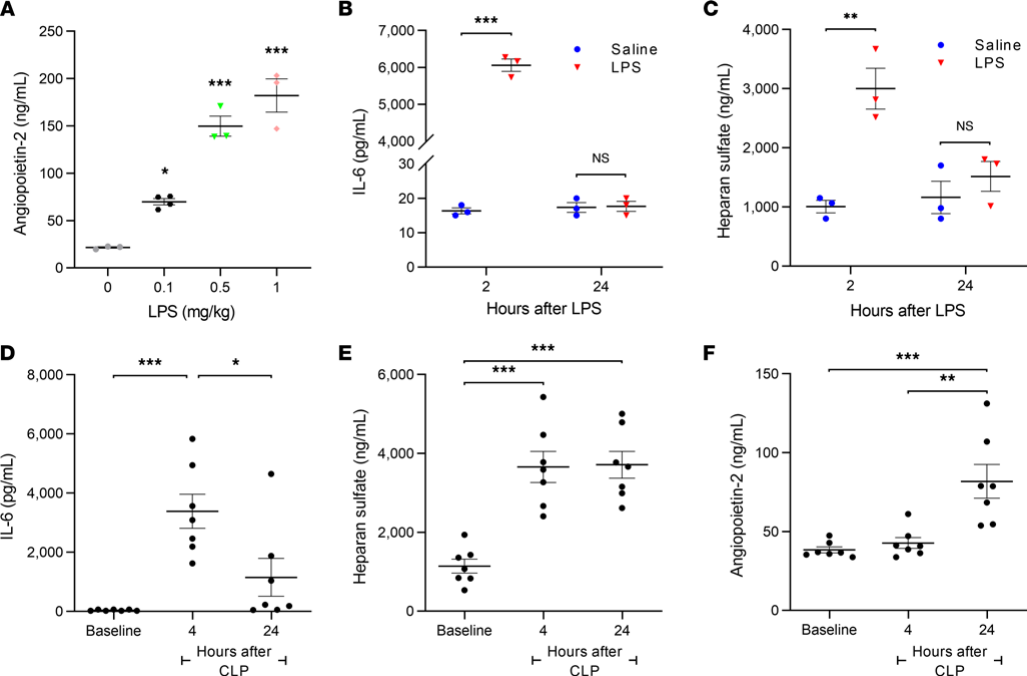
Elevations in plasma HS levels rise before plasma Ang-2 in mice with sepsis. (**A**) Twenty-four-hour plasma levels of Ang-2 in C57BL/6J WT 12- to 14-week-old male mice (*n* = 3–4 per group) following retro-orbital injection with lipopolysaccharide (LPS) 0.1, 0.5, 1 mg/kg or vehicle (saline). *P < 0.05 and ***P < 0.001, versus vehicle. (**B**) IL-6 and (**C**) HS levels in mouse plasma 2 and 24 hours after either a sublethal injection of LPS 0.1 mg/kg or injection with equivalent volume of sterile saline 0.9% (*n* = 3 per group). **P < 0.01, ***P < 0.001. (**D**) IL-6, (**E**) HS, and (**F**) Ang-2 levels in plasma from C57BL/6J WT 12- to 14-week-old male mice at baseline (24 hours prior to cecal ligation and puncture, or CLP) and 4 hours and 24 hours following CLP (*n* = 7). *P < 0.05, **P < 0.01, ***P < 0.001. All data are presented as mean ± SEM and analyzed by ordinary (**A**) or repeated measures (**D**–**F**) 1-way ANOVA corrected by Tukey’s multiple comparisons test or by Student’s 2-sided *t* test (**B** and **C**).

To clarify the temporal relationship between the onset of inflammation, HS cleavage, and elevation in Ang-2 plasma levels during sepsis, we measured plasma levels of IL-6, HS, and Ang-2 using murine models of sepsis. Using a lipopolysaccharide (LPS) model for Gram-negative sepsis (Supplemental Figure 1A; supplemental material available online with this article; https://doi.org/10.1172/jci.insight.155010DS1), we observed that plasma Ang-2 levels in mice rose after LPS injection in a dose-dependent manner (Figure 2A) but did not elevate significantly from baseline levels until 24 hours after injection (Supplemental Figure 1B). However, there was a significant increase in plasma levels of IL-6 and HS by 2 hours following LPS injection (Figure 2, B and C), indicating inflammatory activation and HS cleavage, respectively. We similarly observed that plasma levels of IL-6 and HS rose before significant elevations in plasma Ang-2 levels following cecal ligation and puncture (CLP) (Figure 2, D–F). Together, these data suggest that the inflammatory burst and concurrent HS injury temporally precede endothelial upregulation/release of Ang-2.

### Shear stress results in HS redistribution, cytoskeletal alignment, β-catenin enhancement, and lower ANG2 mRNA expression in HLMVECs.

**Figure 3:**
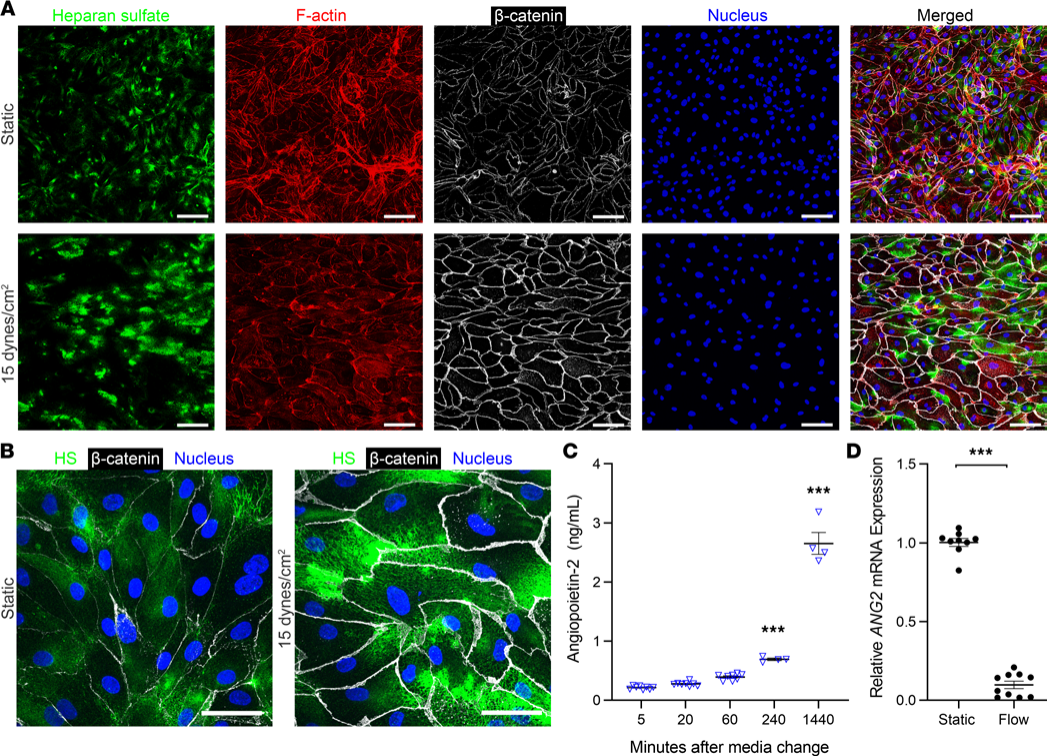
Flow conditioning HLMVECs alters HS distribution and suppresses *ANG2* mRNA expression. (**A**) Stacked 20× original magnification confocal micrographs demonstrating staining for HS (10E4 epitope), F-actin, β-catenin, and cell nuclei (DAPI) on or in HLMVECs after 48 hours of static culture (top row) or 15 dyn/cm^2^ (bottom row). Scale bar: 100 μm. (**B**) Stacked 60× original magnification confocal micrographs demonstrating HS distribution relative to cell edges (demarcated by β-catenin) on HLMVECs after 48 hours of static culture (left panel) or 15 dyn/cm^2^ (right panel) (with DAPI overlay). Scale bar: 20 μm. (**C**) Supernatant Ang-2 levels (ELISA) from a confluent monolayer of HLMVECs over the course of 24 hours of static culture following media change (*n* = 4–8 per group). ***P < 0.001, versus 5 minutes. (**D**) Relative HLMVEC *ANG2* gene expression following 48 hours of either static culture (*n* = 9) or 15 dyn/cm^2^ (*n* = 10). ***P < 0.001. All data are presented as mean ± SEM and analyzed by 1-way ANOVA corrected by Tukey’s multiple comparisons test (**C**) or by Student’s 2-sided *t* test (**D**).

To determine whether HLMVECs used in subsequent mechanistic studies exhibit similar characteristics to other endothelial cells under shear stress (25–27), we evaluated HS distribution within the EG, F-actin cytoskeletal alignment, and adherens junction β-catenin expression in HLMVECs after flow conditioning (15 dyn/cm^2^, 48 hours) compared with HLMVECs in static culture. Similar to work in rat fat pad endothelial cells (15 dyn/cm^2^, 10–30 minutes) (25), shear stress application to HLMVECs increased HS clustering (Figure 3, A and B), particularly at the leading edges of the cell. As with flow-conditioned porcine aortic endothelial cells (15 dyn/cm^2^, 48 hours) (26), F-actin appeared to align in the direction of flow, and β-catenin expression increased at the periphery of flow-conditioned HLMVECs (Figure 3A). We also found that HLMVECs constitutively secreted Ang-2 in static culture (Figure 3C) similar to HUVECs (Supplemental Figure 2A) and human blood and lymphatic endothelial cells (28). Flow conditioning HLMVECs nearly abrogated HLMVEC *ANG2* gene expression (Figure 3D) similar to HUVECs (Supplemental Figure 2B) (21, 29).

### Surface layer HS is integral in regulating Ang-2 expression in flow-conditioned HLMVECs.

**Figure 4:**
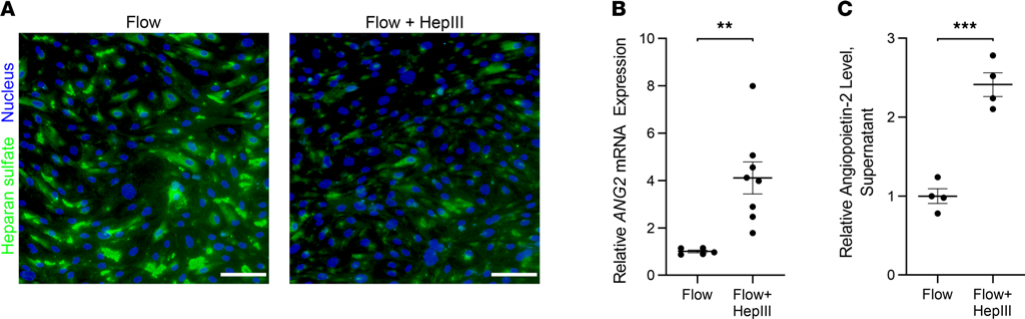
HS over the apical surface of HLMVECs is instrumental in regulating *ANG2* gene and Ang-2 protein expression during flow conditions. (**A**) Representative 20× original magnification epifluorescence images of HS staining (10E4 epitope) on flow-conditioned HLMVECs (15 dyn/cm^2^, 48 hours) followed by an additional 4 hours of 15 dyn/cm^2^ exposure in the absence (left panel) or presence (right panel) of HepIII 200 mU/mL (with DAPI overlay). Scale bar: 100 μm. (**B**) Relative *ANG2* gene expression in flow-conditioned HLMVECs (15 dyn/cm^2^, 48 hours) exposed to 15 dyn/cm^2^ for an additional 24 hours in the absence or presence of HepIII 200 mU/mL (*n* = 5–8 per group). **P < 0.01. (**C**) Relative supernatant Ang-2 levels (ELISA) from flow-conditioned HLMVECs (15 dyn/cm^2^, 48 hours) exposed to 15 dyn/cm^2^ for an additional 24 hours in the absence or presence of HepIII 200 mU/mL (*n* = 4 per group). ***P < 0.001. All data are presented as mean ± SEM and analyzed by Student’s 2-sided *t* test (**B** and **C**).

To determine the role of HS in the regulation of Ang-2 expression from HLMVECs, Bacteroides-derived heparinase III (HepIII) was used to selectively cleave HS from the EG in static and flow conditions. The benefit of using HepIII over recombinant human heparanase lies in its ability to perform its enzymatic function at a neutral pH, unlike mammalian heparanase that requires a more acidic environment that is much less suitable for cell culture (30). Two-hour treatment of statically cultured HLMVECs with HepIII produced a dose-dependent erosion of extracellular HS with a visible increase in staining for the exposed HS 3G10 epitope at a HepIII concentration of 200 mU/mL (Supplemental Figure 3A), suggesting 200 mU/mL as a suitable treatment dose for experimentation. We confirmed enzymatic activity of HepIII under flow conditions, demonstrating that 4-hour treatment of flow-conditioned HLMVECs with HepIII 200 mU/mL resulted in a visible decrease in intact HS (Figure 4A).

Twenty-four-hour treatment of statically cultured HLMVECs with increasing doses of HepIII (200, 500, and 1800 mU/mL) did not alter supernatant Ang-2 levels (Supplemental Figure 3, B and C). We also did not observe significant changes in *ANG2* gene expression following 24-hour HepIII application (200 mU/mL) to statically cultured HLMVECs (Supplemental Figure 3D). Conversely, we found that 24-hour HepIII treatment (200 mU/mL) of flow-conditioned HLMVECs significantly increased levels of *ANG2* mRNA and supernatant Ang-2 protein levels (Figure 4, B and C). These findings together suggest that HS has an important role in shear stress mechanotransduction in HLMVECs and that HS cleavage from the HLMVEC surface contributes to Ang-2 upregulation.

### HS cleavage from flow-conditioned HLMVECs results in decreased AMPK activity that may contribute to increased Ang-2 protein expression.

**Figure 5:**
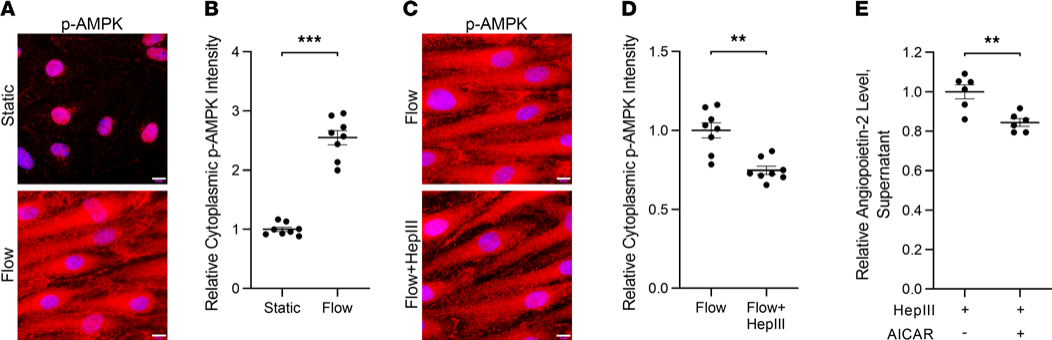
HS cleavage from flow-conditioned HLMVECs decreases shear stress–induced p-AMPK, contributing to Ang-2 secretion. (**A**) Representative 100× original magnification epifluorescence images of HLMVECs stained for p-AMPK following 48 hours of static culture (top) or 15 dyn/cm^2^ (bottom) (with DAPI overlay). Scale bar: 10 μm. (**B**) Relative staining intensity for p-AMPK within the cytoplasm of HLMVECs in static culture or following 15 dyn/cm^2^ (from 8 representative 40× original magnification images, normalized to number of nuclei within field of view). ***P < 0.001. (**C**) Representative 100× epifluorescence images of p-AMPK staining within flow-conditioned HLMVECs (15 dyn/cm^2^, 48 hours) exposed to 15 dyn/cm^2^ for an additional 24 hours in the absence (top) or presence (bottom) of HepIII 200 mU/mL (with DAPI overlay). Scale bar: 10 μm. (**D**) Relative staining intensity for p-AMPK within the cytoplasm of flow-conditioned HLMVECs (15 dyn/cm^2^, 48 hours) exposed to 15 dyn/cm^2^ for an additional 24 hours in the absence or presence of HepIII 200 mU/mL (from 8 representative 40× original magnification images, normalized to number of DAPI-stained nuclei within field of view). **P < 0.01. (**E**) Relative supernatant Ang-2 levels (ELISA) from flow-conditioned HLMVECs (15 dyn/cm^2^, 48 hours) exposed to 15 dyn/cm^2^ and HepIII 200 mU/mL for 24 hours in the presence or absence of AICAR 1 mM. **P < 0.01. All data are presented as mean ± SEM and analyzed by Student’s 2-sided *t* test (**B**, **D**, and **E**).

Prior work demonstrates that shear stress suppresses Ang-2 protein expression from HUVECs through AMPK signaling (21). We similarly observed that flow conditioning HLMVECs significantly increased AMPK phosphorylation (p-AMPK), an indicator of AMPK activation (22), within the cytoplasm of HLMVECs (Figure 5, A and B). We also observed a dose-dependent decrease in supernatant Ang-2 levels from both HLMVECs and HUVECs (Supplemental Figure 4, A and B) following 24-hour treatment with the AMPK activator N1-(β-d-ribofuranosyl)-5-aminoimidazole-4-carboxamide (AICAR). As we observed that shear stress application suppressed *ANG2* gene expression in HLMVECs, together these data support a role for AMPK signaling in the regulation of Ang-2 expression in flow-conditioned HLMVECs.

Next, we determined the effect of HS cleavage on AMPK activation in flow-conditioned HLMVECs to more clearly link HS-mediated mechanoregulation of Ang-2 expression through the AMPK signaling axis. Application of HepIII to flow-conditioned HLMVECs consistently attenuated p-AMPK (Figure 5, C and D). Further, we observed that 24-hour treatment of flow-conditioned HLMVECs with AICAR 1 mM in the presence of HepIII decreased the rise in supernatant Ang-2 levels by 16% (Figure 5E). Together, these findings suggest that increased Ang-2 expression in flow-conditioned HLMVECs that results from surface HS damage occurs, at least in part, through attenuated AMPK signaling.

### Depressed AMPK signaling following HS cleavage results in increased ANG2 expression from flow-conditioned HLMVECs through FoxO1 activation.

**Figure 6:**
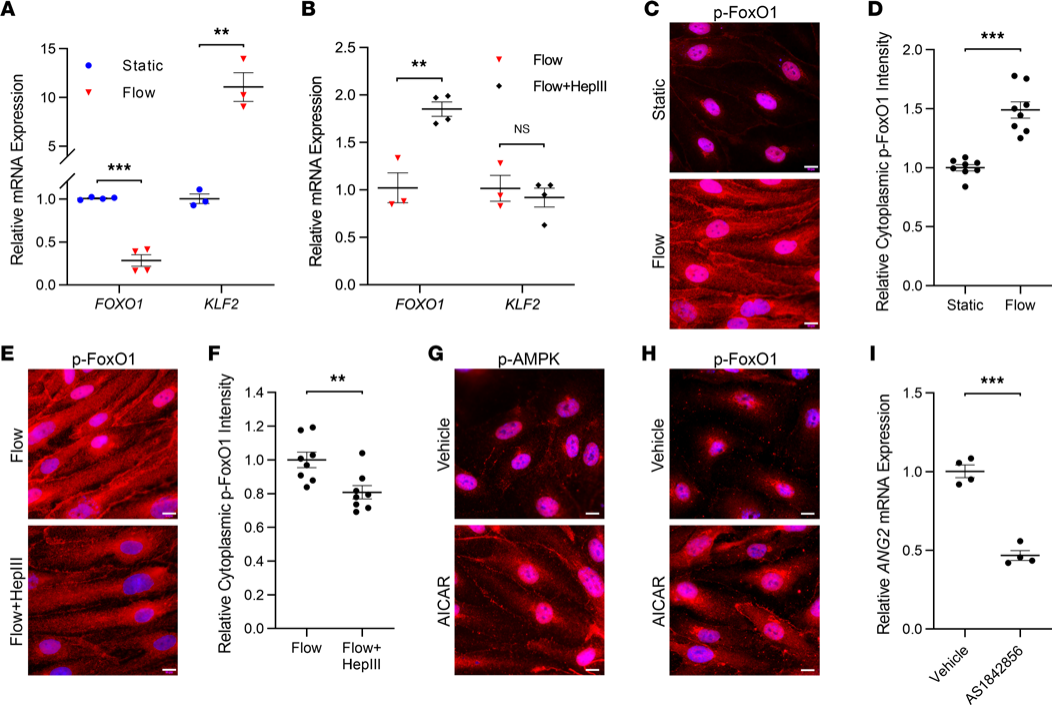
Decreased AMPK activity following HS cleavage from flow-conditioned HLMVECs may increase Ang-2 expression through FoxO1 activation. (**A**) Relative *FOXO1* and *KLF2* gene expression in HLMVECs following 48 hours of either static culture or 15 dyn/cm^2^ (*n* = 3–4 per group). **P < 0.01, ***P < 0.001. (**B**) Relative *FOXO1* and *KLF2* gene expression in flow-conditioned HLMVECs (15 dyn/cm^2^, 48 hours) exposed to an additional 24 hours of 15 dyn/cm^2^ in the absence or presence of HepIII 200 mU/mL (*n* = 3–4 per group). **P < 0.01. (**C**) Representative 100× original magnification epifluorescence images of HLMVECs stained for p-FoxO1 following 48 hours of static culture (top) or 15 dyn/cm^2^ (bottom) (with DAPI overlay). Scale bar: 10 μm. (**D**) Relative staining intensity for p-FoxO1 in HLMVEC cytoplasm following static culture or flow conditioning (from 8 representative 40× original magnification images, normalized to number of nuclei within field of view). ***P < 0.001. (**E**) Representative 100× original magnification epifluorescence images of p-FoxO1 staining in flow-conditioned HLMVECs exposed to an additional 24 hours of 15 dyn/cm^2^ in the absence (top) or presence (bottom) of HepIII 200 mU/mL (with DAPI overlay). Scale bar: 10 μm. (**F**) Relative staining intensity for p-FoxO1 in the cytoplasm of flow-conditioned HLMVECs exposed to an additional 24 hours of 15 dyn/cm^2^ in the absence or presence of HepIII 200 mU/mL (from 8 representative 40× original magnification images, normalized to number of nuclei within field of view). **P < 0.01. Representative 100× original magnification epifluorescence images of p-AMPK (**G**) and p-FoxO1 (**H**) staining in statically cultured HLMVECs treated with AICAR 1 mM or vehicle for 24 hours (with DAPI overlay). Scale bar: 10 μm. (**I**) Relative *ANG2* gene expression in statically cultured HLMVECs treated with the FoxO1 inhibitor AS1842856 or vehicle for 24 hours (*n* = 4 per group). ***P < 0.001. All data are presented as mean ± SEM and analyzed by Student’s 2-sided *t* test (**A**, **B**, **D**, **F**, and **I**).

FoxO1 is a transcriptional activator and KLF2 a suppressor of endothelial *ANG2* gene expression, both of which have been shown to be downstream of flow-activated AMPK signaling (21–24, 29). However, the effect of HS cleavage from flow-conditioned endothelial cells on FoxO1 and KLF2 is wholly unknown. We therefore first evaluated the impact of shear stress on HLMVEC *FOXO1* and *KLF2* gene expression in the presence or absence of HepIII. We found that flow conditioning significantly attenuated *FOXO1* mRNA while increasing *KLF2* mRNA expression (Figure 6A). Though HS degradation substantially increased *FOXO1* mRNA expression within flow-conditioned HLMVECs, we did not observe a change in *KLF2* mRNA following HepIII treatment (Figure 6B).

AMPK regulates FoxO1 transcriptional activity of *ANG2* by dictating FoxO1 phosphorylation (p-FoxO1) that results in FoxO1 transport out of the nucleus to the cytoplasm (21). Therefore, as we observed that HS cleavage from flow-conditioned HLMVECs attenuated p-AMPK that would suggest diminished AMPK activation (22), we next determined the effect of enzymatic HS degradation on the intensity of p-FoxO1 within the cytoplasm of HLMVECs as an indicator of FoxO1 inactivation. As expected, we found that cytoplasmic p-FoxO1 substantially increased following shear stress application (Figure 6, C and D). We also observed that treatment of flow-conditioned HLMVECs with HepIII led to an attenuation of cytoplasmic p-FoxO1 relative to flow conditioning alone (Figure 6, E and F). To confirm that the AMPK/FoxO1 signaling axis contributes to the regulation of Ang-2 expression within HLMVECs, we determined the effect of AMPK activation on p-FoxO1. We observed that 24-hour treatment of statically cultured HLMVECs with AICAR 1 mM resulted in increased fluorescence intensities of both p-AMPK and p-FoxO1 in the cell cytoplasm (Figure 6, G and H) similar to flow conditioning, suggesting that p-FoxO1 is indeed downstream of active AMPK. We then treated HLMVECs with the small molecule AS1842856 to inhibit FoxO1 transcriptional activity (31, 32) and confirmed that FoxO1 regulated *ANG2* gene expression within HLMVECs. We found that FoxO1 inhibition attenuated *ANG2* mRNA expression by nearly 50% (Figure 6I). Taken together, these data support the AMPK/FoxO1 signaling axis as contributory to the regulation of *ANG2* expression from flow-conditioned HLMVECs following surface HS damage.

Finally, we further investigated the AMPK/KLF2 signaling axis by determining whether suppression of KLF2 activity may play a role in AMPK-mediated upregulation of *ANG2* in HLMVECs following HS erosion. Prior work demonstrates that KLF2 transcriptional activity within HUVECs is suppressed by histone deacetylase 5 (HDAC5) during static conditions secondary to cytoplasmic HDAC5 dephosphorylation, subsequent movement into the nucleus, and direct inhibition of KLF2 (33, 34). Application of laminar shear stress (12 dyn/cm^2^, 1–4 hours) was shown to decrease HDAC5 nuclear localization and KLF2 binding (33). Therefore, we investigated whether changes in HDAC5 nuclear localization occur, which could suppress KLF2 activity and result in *ANG2* upregulation following HS damage irrespective of unchanged *KLF2* gene expression (as shown in Figure 6B). However, we observed no significant changes in HDAC5 cell localization following HepIII application (Supplemental Figure 5, A and B). Therefore, given that no changes were observed in either *KLF2* gene expression (Figure 6B) or nuclear localization of KLF2-hindering HDAC5 (Supplemental Figure 5), we conclude that variations in *KLF2* mRNA levels or KLF2 activity do not explain alterations in *ANG2* expression in flow-conditioned HLMVECs following HS injury.

## Discussion

**Figure 7:**
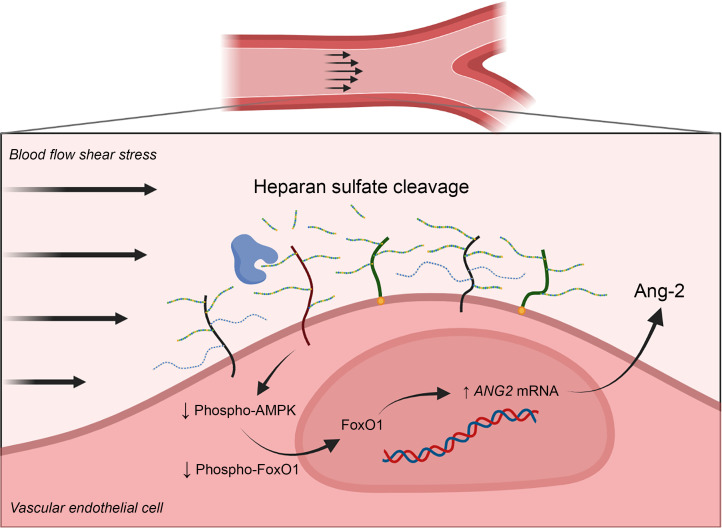
Proposed mechanism by which HS cleavage from the EG during sepsis decreases AMPK signaling, thereby promoting Ang-2 expression. Figure generated using BioRender software.

The vascular endothelium is a critical regulator of blood vessel permeability, inflammation, coagulation, and vascular tone during sepsis (35). Ang-2 is secreted from pathologically activated endothelial cells and may be a key driver of vascular hyperpermeability, endothelial inflammation, and organ dysfunction during sepsis (5, 36). However, the mechanisms driving Ang-2 expression from the endothelium during sepsis remain unknown. In this study, we discovered that HS injury preceded Ang-2 release into circulation in murine models of sepsis, a finding supported by biomarker kinetics in critically ill children with sepsis. We also uncovered that HS cleavage from the EG promoted Ang-2 expression in flow-conditioned HLMVECs by impairing AMPK activity, leading to unhindered FoxO1 transcriptional activation. These findings establish a potentially novel signaling paradigm by which Ang-2 expression may be upregulated by the vascular endothelium during sepsis via loss of HS-mediated mechanotransduction mechanisms (Figure 7).

HS, the most ubiquitous proteoglycan-linked glycosaminoglycan on the apical surface of vascular endothelial cells, is essential for the normal transduction of surface layer shear stress to the endothelial intracellular environment (37–39). Enzymatic cleavage of HS has been shown to attenuate homeostatic nitric oxide (NO) signaling in HUVECs (15, 40). However, to our knowledge, the impact of HS damage on other endothelial signaling pathways, and more specifically on pathways regulating Ang-2 expression, has not been evaluated. Heparanase 1 (or, more commonly, heparanase) is presumed to be the only mammalian endoglucuronidase with enzymatic activity on HS side chains of HSPGs (41, 42). We observed that heparanase activity is significantly higher in the plasma of children with sepsis than healthy controls, similar to adults with sepsis (17–19). We also found that increased circulating levels of HS preceded increases in plasma Ang-2 in murine models of sepsis, suggesting that, in light of the role of HS in transducing endothelial surface layer shear stress, HS cleavage from the EG may contribute to vascular pathobiology through Ang-2 upregulation. In primary HLMVECs, we observed that flow conditioning nearly abrogated *ANG2* mRNA expression while enzymatic HS injury caused significant elevations in both *ANG2* mRNA and protein expression. Interestingly, we did not observe changes in *ANG2* mRNA or protein levels following HS damage in statically cultured HLMVECs, confirming that regulation of constitutive Ang-2 expression in HLMVECs occurs through a mechanism involving HS-mediated mechanosignaling under requisite conditions of shear stress.

Prior work suggests that the shear-mediated regulation of constitutive Ang-2 expression occurs through the AMPK/FoxO1/KLF2 signaling axis (21, 22, 29). However, because these previous studies were performed in HUVECs, it is unknown whether this signaling axis also regulates Ang-2 expression in other human primary endothelial cell types. Using primary HLMVECs, we similarly observed that unidirectional shear stress application (15 dyn/cm^2^, 48 hours) increased p-AMPK and p-FoxO1 and *KLF2* mRNA expression in concert with an attenuation of *FOXO1* and *ANG2* mRNA expression. Further, we found that small molecule activation of AMPK increased p-FoxO1 and that small molecule inhibition of FoxO1 decreased *ANG2* mRNA expression in HLMVECs. Together, these data suggest that shear stress application to HLMVECs activates AMPK that, in turn, promotes KLF2 and inhibits FoxO1, leading to decreased *ANG2* gene expression. We also observed that injury to HS on flow-conditioned HLMVECs decreased p-AMPK and p-FoxO1 while increasing *FOXO1* mRNA expression without an effect on *KLF2* expression. Moreover, we found that AICAR-induced AMPK activation within HepIII-treated flow-conditioned HLMVECs attenuated supernatant levels of Ang-2. Therefore, we provide evidence that AMPK/FoxO1 signaling may dictate Ang-2 expression in human primary endothelial cells other than HUVECs during flow conditions and that damage to HS in the endothelial surface layer promotes aberrant AMPK/FoxO1 signaling that may contribute to pathologic Ang-2 expression.

Though our findings suggest that depressed AMPK/FoxO1 signaling contributes to Ang-2 upregulation following HS cleavage from flow-conditioned endothelial cells, the partial suppression of Ang-2 expression from HepIII-treated HLMVECs by AICAR suggests that other signaling axes are likely at play. Increased endothelial Ang-2 expression involves upregulation of both gene expression and protein secretion. Various signaling pathways have been linked to both mechanisms. PI3K/Akt signaling has previously been shown to suppress constitutive *ANG2* mRNA expression in flow-conditioned HUVECs (6 dyn/cm^2^, 24 hours) with evidence that this may occur through p-FoxO1 and nuclear exclusion (29). However, work by Dixit et al. (21) challenges this particular mechanism as impairment of Akt signaling did not result in changes in p-FoxO1 within flow-conditioned HUVECs (12 dyn/cm^2^, 30 minutes). The PI3K/Akt pathway may regulate endothelial Ang-2 secretion by increasing both expression and activity of endothelial NO synthase (eNOS) (43–45), resulting in increased intracellular levels of NO that inhibit Ang-2 release (44). Using bovine aortic endothelial cells conditioned with 6 hours of either 5 dyn/cm^2^ laminar shear stress or 10 dyn/cm^2^ shear stress under pulsatile flow (1 Hz), Zhang et al. (46) showed that eNOS activity may also be controlled by AMPK signaling. Together, these studies point to multiple, perhaps redundant, mechanisms directing eNOS activity that, through NO signaling, may control Ang-2 secretion. Florian et al. (15) demonstrated that HS cleavage with HepIII 15 mU/mL attenuated NO generation from bovine aortic endothelial cells only in conditions of flow (20 dyn/cm^2^, 3 hours) and not in static culture, a finding later confirmed in vivo (39). In light of our observation that damage to endothelial surface HS resulted in increased Ang-2 expression only during flow conditions, these findings suggest that pathways regulating Ang-2 expression other than AMPK/FoxO1 may also be regulated by HS-mediated mechanosignaling. However, further work is needed to clarify the relative contributions of each of these and other signaling pathways in regulating endothelial *ANG2* gene expression and/or protein release after HS digestion during flow conditions.

Given the impact of HS injury in lung disease observed in sepsis (17) and the associations between Ang-2 and lung dysfunction in sepsis (36) and other critical illnesses (47, 48), we performed our investigations using primary HLMVECs. Furthermore, we conditioned cells with laminar shear stress of 15 dyn/cm^2^ for 48 hours to more closely mimic microvascular blood flow. However, we acknowledge that wide variations in microvascular shear stress within various tissues have been reported (e.g., range of 2.8–95 dyn/cm^2^ in human conjunctival capillaries with average of 15.4 dyn/cm^2^ in ref. 49; 20 dyn/cm^2^ in cat mesenteric capillaries in ref. 50; 5–23 dyn/cm^2^ in rat brain capillaries in ref. 51; 3–12 dyn/cm^2^ in rat mesenteric venular microvessels in ref. 52). Moreover, to our knowledge, magnitude of shear stress within human pulmonary microvasculature has not been directly measured. We also recognize that microvascular blood flow distribution throughout the lung is heterogenous, likely translating into significant variability in shear stress magnitudes throughout the lung. Therefore, it is unknown how precisely our current simplified model recapitulates microvascular fluid dynamics within the human lung. Similar to HLMVECs conditioned with 15 dyn/cm^2^ for 48 hours, we observed increased p-AMPK and *KLF2* mRNA expression along with decreased *FOXO1* and *ANG2* mRNA expression from HLMVECs conditioned with 10 dyn/cm^2^ for 48 hours (Supplemental Figure 6). Given the limitations of our current flow model, we were unable to examine the impact of shear stress lower than 10 dyn/cm^2^. Further studies are planned to evaluate the impact of HS cleavage on Ang-2 expression from HLMVECs under a wider range of shear stress. Moreover, investigations are needed to evaluate the importance of HS/AMPK/FoxO1 signaling in regulating Ang-2 expression within nonpulmonary human primary endothelial cells that may inform future in vivo studies.

This work offers insights into the vascular derangements occurring in sepsis and has implications for other infectious disease processes (e.g., disseminated herpes simplex virus or dengue viral infection) and for noninfectious processes (e.g., ischemia/reperfusion injury, atherosclerosis, cancer dissemination) where heparanase may have a prominent role in pathogenesis (42, 53–57). Our findings are particularly relevant in this day and age of the severe acute respiratory syndrome coronavirus 2 pandemic. Endotheliopathy has become recognized as a central pathophysiologic driver of the nonpulmonary manifestations of severe coronavirus disease 2019 (COVID-19) (58–60). Interestingly, heparanase activity and HS levels are elevated in adults hospitalized with severe COVID-19 similar to patients with sepsis (61). In a separate adult cohort, patients with severe COVID-19 were found to have significantly higher plasma levels of Ang-2 compared with healthy adults or adults with less severe COVID-19 (62). Therefore, our findings that link EG HS erosion to Ang-2 expression may also have implications in the pathogenesis of severe COVID-19, warranting future investigation.

There are a number of important limitations to this study. The flow model we used does not allow for sufficient levels of cell protein to be collected for quality immunoblotting, limiting our analyses to changes in intracellular immunofluorescence patterns. Though we took careful precaution to adjust immunofluorescence of phosphorylated proteins for cell density, we were unable to normalize to the fluorescence intensity of total respective protein as would be performed with Western blot. This limitation may therefore lead to overestimation of the level of protein phosphorylation relative to the quantity of total protein present. Given the higher flows required to maintain consistent shear stress application within our 4-channel flow model, the model is limited to the application of a minimum shear stress of 10 dyn/cm^2^. While the application of 10–15 dyn/cm^2^ arguably mimics in vivo microvascular conditions (49, 63, 64), we were unable to evaluate the effect of HS injury on Ang-2 expression from HLMVECs in shear stress conditions below 10 dyn/cm^2^. In vitro experiments were also performed using HLMVECs from a single donor, restricting the applicability of our findings to other endothelial cell types and the generalizability of our data to all HLMVECs. We also cannot definitively state whether HS cleavage resulted in predominantly increased *ANG2* gene expression, Ang-2 protein release, or a combination of the two. Finally, our study did not consider the potential effects of shed HS fragments on autocrine/paracrine signaling mechanisms that may have contributed to Ang-2 regulation. Given that soluble fragments of the degraded EG have recently been shown to have important bioactivity (65–67), these considerations are logical for future investigations.

In summary, we have uncovered a discrete endothelial cell signaling axis by which Ang-2 may be upregulated in sepsis following the enzymatic removal of HS from the vascular EG. However, this work requires careful validation in a wider range of human primary endothelial cell types and in further in vivo studies. Our observations provide sufficient premise to continue delving into the mechanisms driving Ang-2 expression following HS damage in the apical EG and open the door to exploring the contributions of other EG components in aberrant endothelial signaling. Through these lines of inquiry, we hope to uncover treatments that will substantially improve vascular dysfunction during sepsis and other states of systemic inflammation by restoring EG integrity and/or attenuating pathologic Ang-2 production.

## Methods

### Materials.

*Escherichia coli*–derived (0111:B4) LPS was purchased from InvivoGen. Bacteroides HepIII was obtained from New England Biolabs. AICAR was purchased from Tocris Bioscience, Bio-Techne. AS1842856 was from MilliporeSigma. Primary antibodies against the HS 10E4 epitope (intact HS) (mouse anti-human monoclonal IgM, clone 8.S.087; catalog H1890) and 3G10 epitope (cleaved HS) (mouse anti-human monoclonal IgG2b, clone 8.S.222; catalog H1890-75) were purchased from United States Biological. Primary antibodies against p-FoxO1 (Ser256) (rabbit anti-human polyclonal IgG; catalog PA5-104977) and p-AMPKα1,2 (Thr183, Thr172) (rabbit anti-human polyclonal IgG; catalog 44-1150G) were obtained from Invitrogen, Thermo Fisher Scientific. Primary antibodies against β-catenin (rabbit anti-human monoclonal IgG, clone E247; catalog ab32572) were from Abcam. Rhodamine phalloidin conjugated to TRITC was obtained from Invitrogen, Thermo Fisher Scientific (catalog R415), to identify F-actin. Alexa Fluor 488–labeled secondary antibodies (goat anti-mouse IgM, IgG; catalog A10680) were obtained from Invitrogen, Thermo Fisher Scientific. DyLight 594–labeled secondary antibodies (goat anti-rabbit IgG; catalog ab96885) were obtained from Abcam. Alexa Fluor Plus 647–labeled antibodies (goat anti-rabbit IgG; catalog A32733) were from Invitrogen, Thermo Fisher Scientific.

### Human patients.

We performed a secondary analysis of data collected from a previously published prospective observational study (4). Briefly, 38 children with sepsis admitted to the Children’s of Alabama PICU were enrolled between August 2018 and January 2020. Whole blood was collected into sodium citrate specimen tubes at PICU admission and 24, 48, and 72 hours after admission. Thirty-eight previously healthy children presenting to the Children’s of Alabama outpatient dental rehabilitation clinic were enrolled as controls, and blood specimens were similarly collected in sodium citrate specimen tubes at the time of anesthesia induction. All specimens were processed within 30 minutes, and platelet-poor plasma was aliquoted and stored at –80°C.

### Murine models.

For LPS studies, C57BL/6J WT 12- to 14-week-old male mice (Charles River Laboratories) underwent general anesthesia with isoflurane 2% followed by retro-orbital injection of either LPS or equivalent volumes of sterile saline 0.9% as sham controls. CLP was performed using C57BL/6J WT 12- to 14-week-old male mice after inducing general anesthesia with intraperitoneal injections of ketamine 75 mg/kg and xylazine 10 mg/kg followed by subcutaneous injection of sustained-release buprenorphine 1 mg/kg into the scruff. The cecum was exposed through a 1 cm vertical midline laparotomy, the distal 5 mm was ligated using 4-0 silk suture, and a through-and-through puncture was made in the ligated portion of the cecum using a 22G needle. After a small mound of feces was expressed from each puncture site, the cecum was reinternalized, and the peritoneum was closed in layers. Mice were then resuscitated with subcutaneous injection of sterile saline 0.9% 1 mL into the scruff. Following the respective procedures, mice were allowed access to food and water ad libitum and monitored for signs of distress. One-hundred-microliter blood samples were collected from the retro-orbital venous plexus into heparin-coated capillary tubes at the indicated times after induction with isoflurane 2%.

### Human and murine ELISAs.

Human Ang-2 DuoSet ELISA kit was purchased from R&D Systems, Bio-Techne. Human HS ELISA kit was purchased from AMS Biotechnology. Mouse IL-6 DuoSet ELISA and Ang-2 quantikine ELISA were purchased from R&D Systems, Bio-Techne. Mouse HS ELISA was purchased from LifeSpan Biosciences. All human and murine ELISAs were performed after a single freeze-thaw.

For experiments using statically cultured endothelial cells, absolute Ang-2 levels from supernatant are reported. However, as the flow system uses a substantially greater volume of culture medium, absolute measures of Ang-2 are impossible to interpret against experiments using static conditions. For experiments using flow-conditioned endothelial cells, Ang-2 levels were measured in supernatant sampled immediately following 48 hours of flow conditioning (baseline) and 24 hours after the reported experimental condition (24-hour sample). Absolute differences between baseline and 24-hour levels were then calculated and reported as relative values to the mean of the comparative control level.

### Heparanase activity assay.

Heparanase activity in human plasma samples was determined as described previously (68) using a fluorescence resonance energy transfer-based assay purchased from Cisbio to measure homogeneous time-resolved fluorescence according to manufacturer specifications.

### Cell culture.

HLMVECs harvested from the lung of a single healthy adult donor were purchased from Cell Applications, cultured on surfaces coated with attachment factor solution (AFS, Cell Applications), and maintained in microvascular endothelial cell growth medium (Cell Applications). HUVECs harvested from the umbilical vein of a single healthy neonate were purchased from Cell Applications and maintained in endothelial cell all-in-one growth medium (Cell Applications). All experiments with HLMVECs were performed using passage 4–6 cells. Experiments with HUVECs were performed using passage 3–6 cells. Experimental conditions were maintained in a Heracell VIOS incubator (Thermo Fisher Scientific) using humidified air with 5% CO_2_ at 37°C. For the measurement of constitutive Ang-2 release, HLMVECs and HUVECs were grown in flat-bottom, 96-well microplates (Corning) and media collected at the indicated time points. Samples were centrifuged at 10,000*g* for 10 minutes at 4°C, and harvested supernatant was stored at –80°C. For flow-related experiments, HLMVECs or HUVECs were grown to a confluent monolayer in 0.4 mm height, μ-slide Luer single-channel flow chambers purchased from Ibidi. Endothelial cells were then either maintained in static culture with twice-daily media changes or conditioned under shear stress using a flow system.

### Flow model.

The flow system used for the current study incorporated a Levitronix BPSi30 centrifugal pump head driven by a LabVIEW-based platform (National Instruments) to flow 35 mL of culture medium across 4 parallel, single-channel Ibidi flow chambers in a closed loop. Unidirectional, laminar flow was adjusted within the flow model to maintain uniform shear stress across the 4 flow channels at 10 or 15 dyn/cm^2^ as indicated. Cells that were flow conditioned underwent application of the indicated level of shear stress for 48 hours prior to further treatment.

### Cell culture treatments.

For HepIII-related experiments, indicated concentrations of HepIII were added directly to a confluent monolayer of cells in channel slides maintained in either static culture or the flow system for 2, 4, or 24 hours as indicated. For experiments evaluating the effect of AICAR treatment on supernatant levels of Ang-2, cells were grown in flat-bottom, 96-well microplates and exposed to the indicated AICAR concentrations for 24 hours prior to media collection. For experiments evaluating the effect of AICAR treatment on Ang-2 release during concurrent treatment of flow-conditioned HLMVECs with HepIII, AICAR 1 mM was added directly to the flow system at the same time HepIII treatment was applied. For experiments evaluating the effect of AICAR treatment on AMPK and FoxO1 phosphorylation, HLMVECs were grown to confluence on AFS-treated 12 mm diameter #1 glass coverslips (Neuvitro) in a 24-well culture plate (Corning); cells were then treated with AICAR 1 mM for 24 hours, briefly washed with PBS, and prepared for immunofluorescence. For studies involving the inhibition of FoxO1 transcriptional activity, HLMVECs were grown to confluence in 24-well culture plates and treated with AS1842856 5 μM for 24 hours prior to mRNA collection.

### Immunofluorescence.

Cells in static culture grown to confluence in flow chamber slides were fixed with 4% paraformaldehyde in PBS for 20 minutes at room temperature, whereas cells in a flow environment were dynamically fixed by simultaneously infusing 4% paraformaldehyde while draining media and then allowing paraformaldehyde to perfuse over cells for 20 minutes at room temperature. Slide chambers were designated for surface staining or for intracellular staining. For surface staining, cells were washed and blocked with 2% goat serum for 1 hour. Cells undergoing intracellular staining were washed, permeabilized with Triton X-100 0.1%, and blocked with 2% goat serum for 1 hour. Standard immunocytochemistry was performed by incubating cells with primary antibody (1:100 dilution) overnight at 4°C followed by gentle washing with Tween 20 0.1% and incubation with secondary antibody (1:500) at room temperature in a dark space. Cell nuclei were stained using DAPI (Thermo Fisher Scientific) (1:1000). Fluorescence was captured using an Olympus BX60 fluorescence microscope with identical objective and exposure for respective experimental conditions along the middle portion of the slide, where flow was most laminar. Representative images were recorded using an Olympus DP74 digital camera and Olympus cellSens Dimension imaging software. Semiquantification of fluorescence intensity was performed using ImageJ (1.53c, NIH). Confocal microscopy was performed using a Nikon A1R laser scanning microscope and NIS-Elements imaging software for image capture.

Immunofluorescence intensity changes in the cytoplasmic fraction of p-AMPK or p-FoxO1 were used to indicate level of AMPK activation or FoxO1 inactivation, respectively, due to the limitation of the flow model for cell protein collection to perform Western blot. Cytoplasmic intensities of p-AMPK or p-FoxO1 were calculated by subtracting the fluorescence pixel density within the nuclei from the total image pixel density. The cytoplasmic fluorescence intensity of each protein was then normalized to number of cells within the respective field of view as indicated by number of DAPI-stained nuclei.

### RNA isolation and RT-qPCR.

Total RNA was isolated from cell lysate of HLMVECs grown in flow chamber slides using the Cytiva illustra RNAspin Mini Isolation Kit according to manufacturer protocol (Thermo Fisher Scientific). RNA was converted to cDNA using a high-capacity cDNA reverse transcription kit (Thermo Fisher Scientific), and cDNA underwent PCR using TaqMan gene expression assays for human *ANG2* (assay ID Hs00169867_m1), *FOXO1* (Hs00231106_m1), and *KLF2* (Hs00360439_g1) mRNA (Thermo Fisher Scientific). PCR reactions were quantified using a QuantStudio 6 Flex System from Applied Biosystems, Thermo Fisher Scientific, and gene expression was normalized to glyceraldehyde-3-phosphate dehydrogenase (*GAPDH*) gene expression (Hs02758991_g1, Thermo Fisher Scientific) using the 2^-ΔΔCT^ method.

### Statistics.

Clinical data are presented as median (interquartile range) provided the nonparametric data distribution was evaluated by the Kolmogorov-Smirnov test for normality. Two groups were compared by an unpaired, 2-tailed Mann-Whitney *U* test. More than 2 groups were compared using the Kruskal-Wallis 1-way ANOVA followed by Dunn’s multiple comparison tests. Spearman’s rank-order correlation was used to determine the correlation between plasma levels of HS at admission and plasma Ang-2 levels 24 hours after admission. For experiments, 2 groups were compared with unpaired, 2-sided Student’s *t* tests. More than 2 groups were compared using either ordinary 1-way ANOVA followed by Tukey’s multiple comparisons test or 2-way ANOVA followed by Holm-Šídák multiple comparisons test as appropriate. For serial measurements within mouse experiments, repeated measures 1-way ANOVA was used as indicated with Greenhouse-Geisser correction for sphericity departures followed by Tukey’s multiple comparisons test. A 2-tailed *P* < 0.05 was considered statistically significant. All analyses were performed using GraphPad Prism software, version 9.0.

### Study approval.

The clinical study was approved by the University of Alabama at Birmingham Institutional Review Board (protocol 300001022). All animal studies were performed in accordance with the Animal Welfare Act following University of Alabama at Birmingham Institutional Animal Care and Use Committee approval (protocol 21775).

## Author contributions

RPR, RDS, AG, and JRR designed the project. RPR, ARA, LZ, DP, KT, and JRR performed experiments. RPR, RDS, AG, and JRR provided data analysis and interpretation. RPR and JRR prepared the manuscript. All authors approved the manuscript.

## Supplementary Material

Supplemental data

## Version 1 06/28/2022

In-Press Preview

## Version 2 08/08/2022

Electronic publication
