# Supplementary material for: Glycocalyx heparan sulfate cleavage promotes endothelial cell angiopoietin-2 expression by impairing shear stress–related AMPK/FoxO1 signaling
Source: JCI Insight. 2022 Aug 8;7(15):e155010. doi: 10.1172/jci.insight.155010 (PMC9462499; doi:10.1172/jci.insight.155010)
Supplement: Supplemental data [file jciinsight-7-155010-s053.pdf]

Glycocalyx heparan sulfate cleavage promotes endothelial cell angiopoietin-2 expression by impairing shear stress-related AMPK/FoxO1 signaling

Robert P. Richter, Amit R. Ashtekar, Lei Zheng, Danielle Pretorius, Kaushlendra Tripathi, Ralph D. Sanderson, Amit Gaggar, Jillian R. Richter

SUPPLEMENTAL MATERIAL

FIGURES

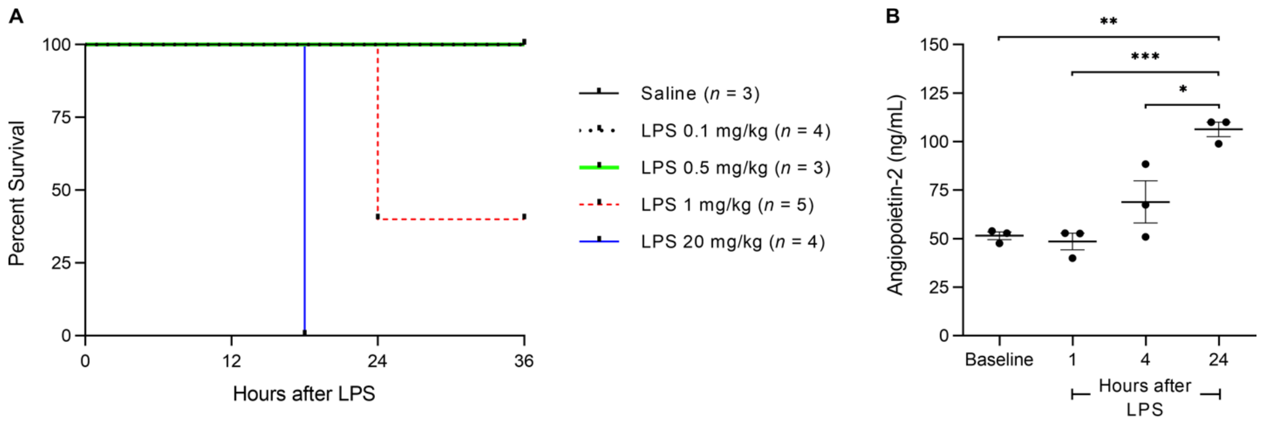

Supplemental Figure S1. Survival curve of increasing doses of lipopolysaccharide (LPS) administration and the time-dependent response of plasma angiopoietin-2 levels following LPS injection. (**A**) Kaplan-Meier survival curves of groups of C57BL/6J WT 12-14 week-old male mice who received retro-orbital injections with LPS 0.1, 0.5, 1, 20 mg/kg or vehicle (n = 3-5). We observed that LPS doses less than or equal to 0.5 mg/kg were sublethal, informing the LPS 0.1 mg/kg dose used to measure plasma levels of IL-6 and heparan sulfate depicted in Figure 2B and 2C. (**B**) Plasma angiopoietin-2 (Ang-2) levels in the three surviving mice following LPS 1 mg/kg injection shown in panel A. *$P < 0.05$, **$P < 0.01$, and ***$P < 0.001$ versus baseline after ordinary 1-way ANOVA corrected by Tukey's multiple comparisons test. Data are presented as mean ± SEM.

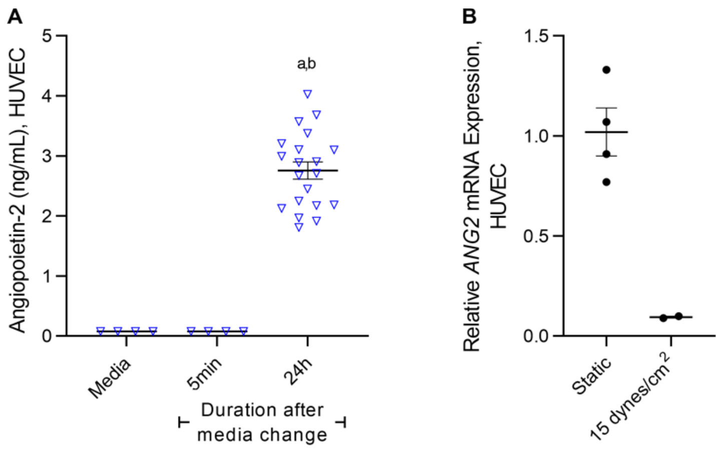

Supplemental Figure S2. Constitutive *ANG2* gene expression in human umbilical vein endothelial cells (HUVEC) is suppressed by shear stress application. (**A**) Ang-2 levels (ELISA) in HUVEC media (n = 4), HUVEC supernatant within 5min of media change (n = 4), and HUVEC supernatant after 24h of static culture (n = 20). $^{a}P < 0.001$ versus media, $^{b}P < 0.001$ versus 5min after ordinary 1-way ANOVA corrected by Tukey's multiple comparisons test. (**B**) Relative HUVEC *ANG2* gene expression after 24h of static culture (n = 4) or 48h of 15 dyn/cm^2 (n = 2). All data are presented as mean ± SEM.

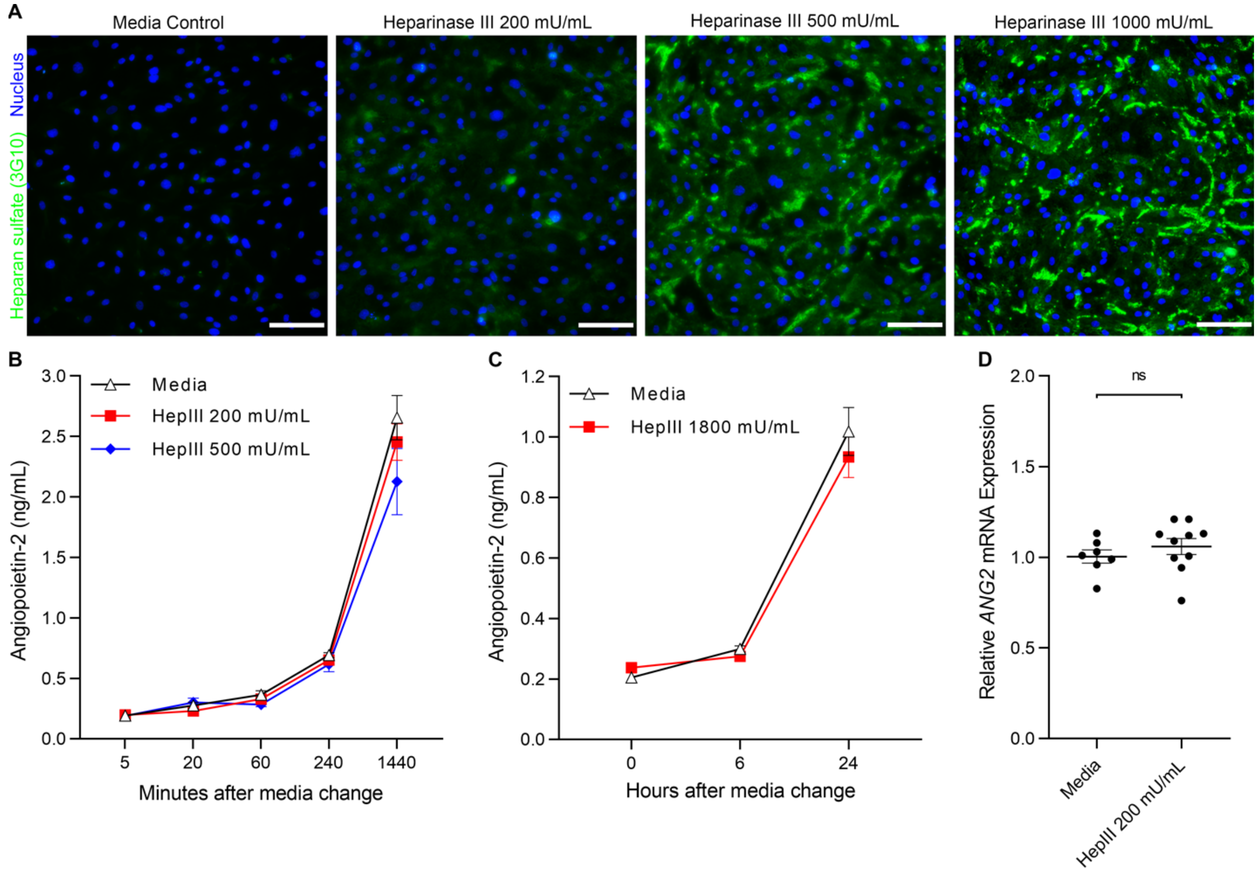

Supplemental Figure S3. Heparinase III (HepIII) treatment of statically cultured human lung microvascular endothelial cells (HLMVEC) results in a dose-dependent erosion of surface heparan sulfate but does not alter Ang-2 protein or *ANG2* gene expression. (**A**) Representative 20x epifluorescence images demonstrating staining for cleaved heparan sulfate (exposed 3G10 epitope) on HLMVECs treated with increasing doses of HepIII (2h treatment) (with DAPI overlay). Scale bar = 100 μm. Note the increased staining intensity for heparan sulfate 3G10 with increasing HepIII concentrations, becoming visually apparent by 200 mU/mL. (**B**) Supernatant Ang-2 levels (ELISA) from HLMVECs over the course of 24h after treatment with vehicle (media), HepIII 200 mU/mL, or HepIII 500 mU/mL (n = 4 per group per time point). There were no significant changes in Ang-2 secretion associated with HepIII dose after 2-way ANOVA corrected by Šídák's multiple comparisons test. (**C**) Supernatant Ang-2 levels (ELISA) from HLMVECs over the course of 24h after treatment with HepIII 1800 mU/mL or vehicle (media) (n = 4 per group per time point). There were no significant changes in Ang-2 levels associated with high-dose HepIII treatment after 2-way ANOVA corrected by Šídák's multiple comparisons test. (**D**) Relative *ANG2* gene expression from statically cultured HLMVECs in the presence (n = 10) or absence (n = 7) of HepIII 200 mU/mL (24h treatment). Ns represents non-significance after Student's *t*-test. All data are presented as mean ± SEM.

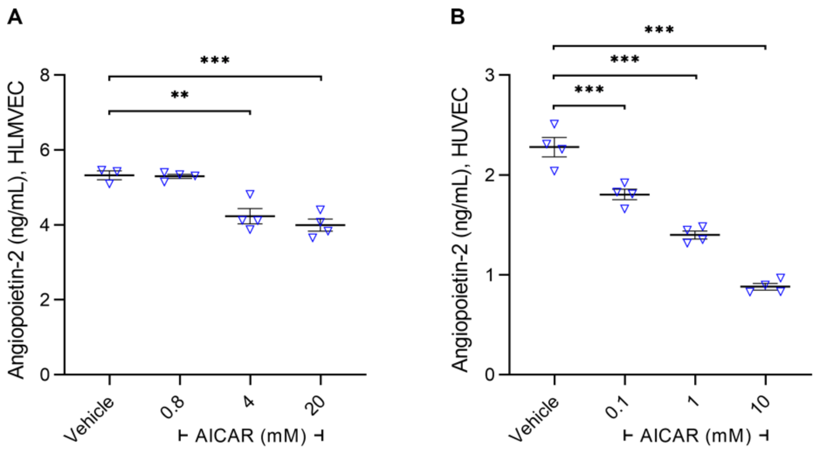

Supplemental Figure S4. Effect of AMPK activation on Ang-2 secretion from HLMVECs and HUVECs. (**A**) Supernatant Ang-2 levels (ELISA) from statically cultured HLMVECs 24h following treatment with increasing doses of AICAR or vehicle (n = 4 per group). ***P* < 0.01, ****P* < 0.001 after 1-way ANOVA corrected by Tukey's multiple comparisons test. (**B**) Supernatant Ang-2 levels (ELISA) from HUVECs 24h after treatment with increasing doses of AICAR or vehicle (n = 4 per group). ****P* < 0.001 after ordinary 1-way ANOVA corrected by Tukey's multiple comparisons test. All data are presented as mean ± SEM.

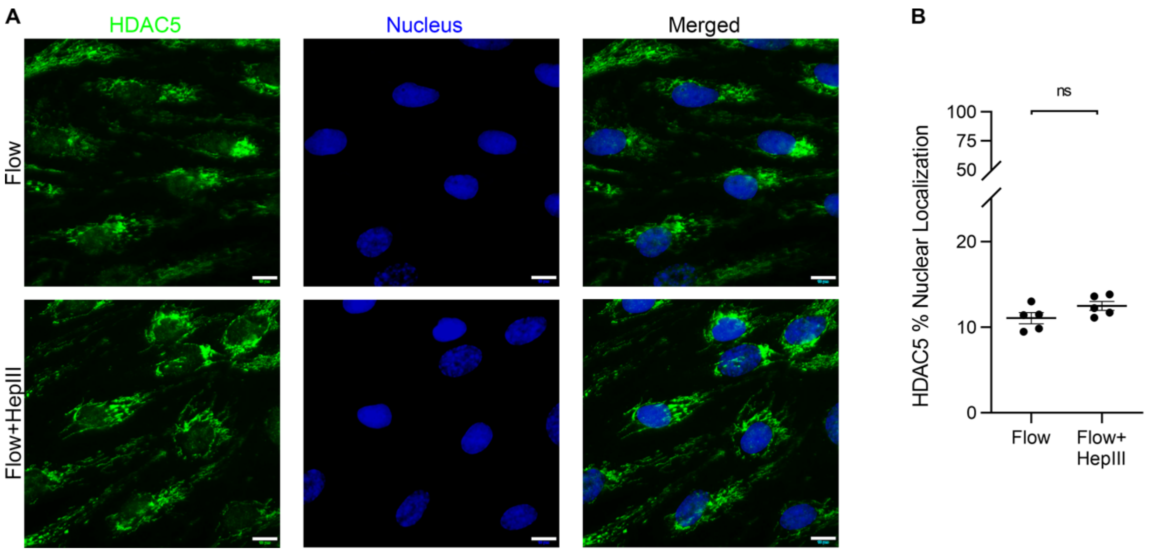

Supplemental Figure S5. Heparan sulfate cleavage from flow conditioned HLMVECs does not affect cellular localization of histone deacetylase 5 (HDAC5). (**A**) Representative 100x epifluorescence images demonstrating staining for HDAC5 in flow conditioned HLMVECs (15 dyn/cm^2, 48h) exposed to an additional 24h of 15 dyn/cm^2 in the absence (top row) or presence (bottom row) of HepIII 200 mU/mL (with DAPI overlay). Scale bar: 10 µm. (**B**) Relative staining intensity for HDAC5 within the nuclear fraction of flow conditioned HLMVECs (15 dyn/cm^2, 48h) exposed to an additional 24h of 15 dyn/cm^2 in the absence or presence of HepIII 200 mU/mL (from 5 representative 20x images, normalized to number of nuclei within field of view). Ns represents non-significance after Student's *t*-test. Data presented as mean ± SEM.

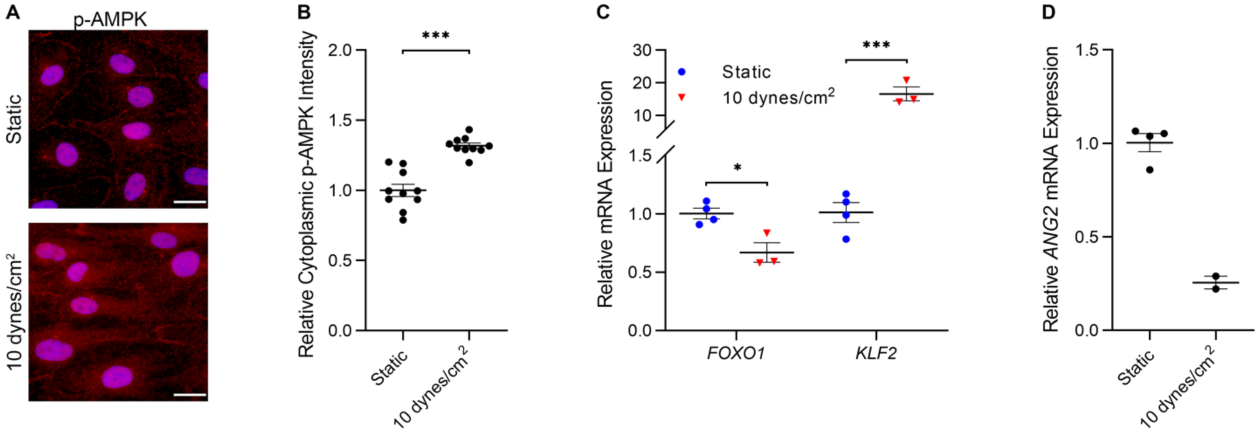

Supplemental Figure S6. Effect of lower shear stress on 5'-adenosine monophosphate-activated protein kinase (AMPK) phosphorylation and Forkhead Box O1 (*FOXO1*), Krüppel-like factor 2 (*KLF2*), and *ANG2* gene expression within HLMVECs. (**A**) Representative 100x epifluorescence images of HLMVECs stained for phosphorylated-AMPK (p-AMPK) following 48h of static culture (top) or 10 dyn/cm^2 (bottom) (with DAPI overlay). Scale bar = 20 µm. (**B**) Relative staining intensity for p-AMPK in the cytoplasm of HLMVECs following 48h of static culture or 10 dyn/cm^2 (from 10 representative 40x images, normalized to number of nuclei within field of view). ***$P < 0.001$ after Student's *t*-test. (**C**) Relative *FOXO1* and *KLF2* mRNA expression in HLMVECs following 48h of static culture (n = 4) or 10 dyn/cm^2 (n = 3). *$P < 0.05$, ***$P < 0.001$ after Student's *t*-test. (**D**) Relative *ANG2* mRNA expression in HLMVECs following 48h of static culture (n = 4) or 10 dyn/cm^2 (n = 2). All data are presented as mean ± SEM.
